# Tensile fracture of a single crack in first-year sea ice

**DOI:** 10.1098/rsta.2017.0346

**Published:** 2018-08-20

**Authors:** J. P. Dempsey, D. M. Cole, S. Wang

**Affiliations:** 1 Department of Civil and Environmental Engineering, Clarkson University, Potsdam, NY 13676, USA; 2 Department of Mechanical Engineering, Aalto University, PO Box 14300, Espoo 00076, Finland; 3 US Army ERDC Cold Regions Research and Engineering Laboratory (Ret.), Hanover, NH 03755, USA; 4 TerraPower LLC, 330 120th Avenue NE STE 100, Bellevue, WA 98005, USA

**Keywords:** fracture, Arctic, Antarctic, stress separation curve

## Abstract

The break-up of sea ice in the Arctic and Antarctic has been studied during three field trips in the spring of 1993 at Resolute, NWT, and the fall of 2001 and 2004 on McMurdo Sound via *in situ* cyclic loading and fracture experiments. In this paper, the back-calculated fracture information necessary to the specification of an accurate viscoelastic fictitious (cohesive) crack model is presented. In particular, the changing shape of the stress separation curve with varying conditions and loading scenarios is revealed.

This article is part of the theme issue ‘Modelling of sea-ice phenomena’.

## 1. Introduction

A fracture mechanics description of the tensile fracture of sea ice is sought. Sea ice mechanics ranges over a vast range in scales [1,2], with topics such as the laboratory-scale failure of ice under compression, structural-scale ice–structure interactions and mesoscale ridging, lead formation and ice rafting. The study of such topics has involved the analysis of subsets of processes based on scale and their interactions with adjacent scales (table 1).

**Table 1.** Nomenclature.

AE	acoustic emission
CMOD	crack-mouth-opening-displacement
CTOD	crack-tip-opening-displacement
COD	crack-opening-displacement
ENSP	edge-notched-square-plate
ERR	energy-release-rate
F1, F2, F3	crack-opening-displacements measured ahead of crack tip
FCM	fictitious crack model
FDPZ	fully developed-process-zone
FPZ	fracture process zone
LEFM	linear elastic fracture mechanics
LVDT	linear variable displacement transducer
NCTOD^−^	near-crack-tip-opening-displacement, before crack-tip
NCTOD^+^	near-crack-tip-opening-displacement, ahead of crack-tip
SIF	stress-intensity-factor
SSC	stress-separation-curve
VFCM	viscoelastic fictitious crack model
Δ*a*	amount of crack growth
*A*_tf_	traction-free crack length
*A*_pz_	length of fracture process zone
*d*_av_	average sea ice grain size
*h*	sea ice thickness
*K*_Q_	apparent fracture toughness
*L*	crack-parallel test size
*ρ*_ i_	sea ice density
S_i_	sea ice salinity
*σ*_t_	local sea ice tensile strength
T	sea ice temperature
*w*_c_	critical crack separation at the trailing edge of a FDPZ

From a reality-based fracture mechanics viewpoint, it is to be appreciated at the outset that cracks in ice nucleate and grow on different planes, on many orientations, from un-cracked surfaces, from within, and propagate from existing crack tips. The sought-after fracture mechanics for sea ice should handle crack formation from an un-notched state, crack propagation from small to large, and the fracture characteristics will differ given different settings (such as *load control* or *CMOD control* and varying stiffness of the loading device).

**Figure 1.**
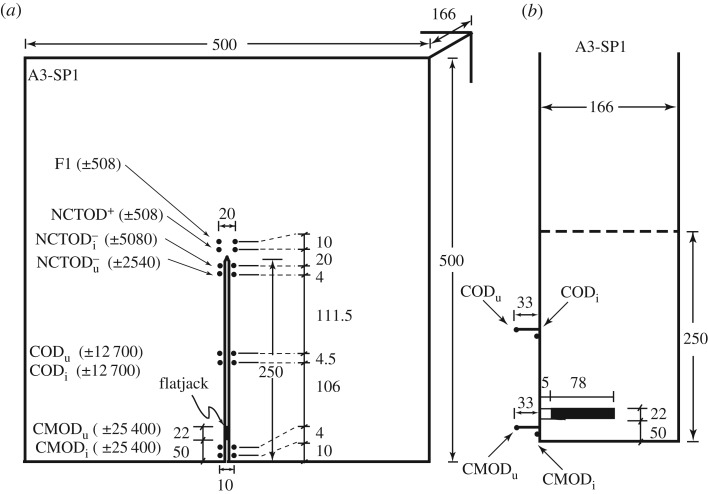
(*a*) Plan view of the test configuration for A3-SP1 (*L* = 5 m, *A* = 2.5 m). (*b*) Side view of the pre-sawn crack showing the flatjack placement. All dimensions are in centimetres; the LVDT ranges (± range) are in microns.

Six field trips to the Arctic were conducted between 1992 and 1994 (labelled as Phase I to Phase VI) [3], and five field trips were conducted in Antarctica between 2001 and 2007 (labelled A1 to A5). This paper will discuss fracture tests conducted in 1993 (Phase II), 2001 (A2) and 2004 (A3). During these field trips, each *in situ* Mode I fracture test involved an edge-notched-square-plate (ENSP) configuration of side-length *L* and crack length *A*. A typical test configuration is shown in figure 1. The fabricated traction-free crack plane was created by a vertical saw cut. The fabricated crack occupied the region 0 ≤ *X* ≤ *A*, *Y* = 0 ± , −*h*/2 ≤ *Z* ≤ *h*/2, where *h* is the sea ice thickness. This type of cracking is described in the ice literature as radial cracking [4,5] or splitting [6]. The crack front lies in the vertical (V) plane parallel to the long axis of the columnar grains, parallel also to the *X*, *Z*-plane, and the crack propagation direction is horizontal (H), this mode of cracking being labelled as VH in fig. 1 of [7]. To be more specific, the vertical crack front occupying *X* = *A*, −*h*/2 ≤ *Z* ≤ *h*/2 is simplistically assumed to propagate with the crack front staying vertical and the crack length increasing monotonically in the *X*-direction within the zone *A* < *X* < *L*.

## 2. An ill-advised one-parameter fracture mechanics ice odyssey

For the chosen ENSP test geometry, given an equal and opposite concentrated load *P* load applied at the crack mouth, and given a process-zone of vanishing size attached to the traction-free crack-tip, the Mode I stress-intensity-factor (SIF) *K*_I_ or energy-release-rate (ERR) *G*_I_ expressions may be written in the generic forms
2.1
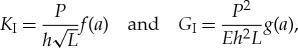
in which *a* = *A*/*L*. For short duration fracture tests, *E* is regarded as the short-time modulus. In metals, any crack opening displacement near the crack front causes a triaxial state of stress in the vicinity of the crack front and dimpling of the side surfaces. This dimpling is used directly by the method of caustics [8]. In quasi-brittle materials (concrete, ice, rock), *σ*_*ZZ*_ is bounded by the local tensile strength *σ*_t_, and cleavage cracks (HH in fig. 1 of [7]) nucleate at the crack front to relieve the stress parallel to the crack front, and this dimpling does not occur (the tensile strength being significantly lower than the compressive strength). In other words, the length of the crack front (*h* in this case) is not expected to influence the fracture energy. This is the reason that, for quasi-brittle materials, regarding the modulus *E*, there is no cause to invoke plane strain.

The typical fracture test programme of yesteryear [9], starting in the early seventies (draft CRREL Technical Note by Liu & Loop, 1972), proceeded as follows (for all types of ice): assume linear-elastic-fracture-mechanics (LEFM), choose a test geometry, choose a test size *L*, cut a traction-free crack of length *A*_0_, let the normalized crack length be *a*_0_ = *A*_0_/*L*, conduct the test in load control, assume unstable fracture occurs at the peak load *P*_max_, evaluate the critical-SIF or critical-ERR, and imperiously label these quantities as *K*_Ic_ and *G*_Ic_, respectively, where
2.2
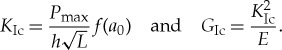


What is the problem with this approach? With the metals-based mindset of yesteryear (still prevalent), the applicability of the ASTM E399 ‘Standard Test Method for Plane-Strain Fracture Toughness of Metallic Materials’ (first tentative version published as last chapter in [10]) was assumed at the outset to apply without validation, and just what the *K*_Ic_ and *G*_Ic_ notation really meant was blithely ignored. A Special ASTM Committee on Fracture Testing (ASTM Committee E-24) was established in 1958 to develop the ASTM E399 Standard. The ASTM E399 standard required testing round-robins, the consideration of many factors [10], such as specimen size requirements: crack length, specimen thickness (length of the crack front), ligament length, specimen geometry independence, notch acuity, and notch sensitivity. The concept of *K*_Ic_ and *G*_Ic_ entails two independent size effects requiring (1) linear elastic behaviour of the material over a field which is large compared with the plastic enclave that surrounds the crack front, and (2) tritensile plane strain constraint within this enclave and somewhat beyond it [11]. The size requirements imposed by ASTM E399 is relevant to high strength metals. ASTM E399 has no established relevance to any type of ice.

Almost every usage of LEFM and the *K*_Ic_ fracture toughness notation in the ice literature indicates ignorance of the fact that LEFM may well not be applicable and that there is no fracture testing standard for any type of ice. One is reminded again and again of the quote: ‘The greater the ignorance the greater the dogmatism’ [12].

During this ill-advised one-parameter fracture mechanics ice odyssey, it has been implicitly assumed that the way a tensile fracture grows in sea ice is universal, from cracks small to large. Given the plethora of fracture scenarios, however, attention must be focused on the physical processes accompanying crack nucleation and crack propagation in sea ice. For instance, during the self-similar ENSP Phase II fracture experiments, it was found that the crack-tip-opening-displacement (CTOD) at the fabricated crack tip varied with specimen size *L* (or, equivalently, with crack length *A*) until the specimen size (crack length) reached 3 m (0.9 m). For *L*≥3 m or *A*≥0.9 m, the CTOD remained a constant 40 μm (fig. 9 and the Non-Universal Scaling section, p. 358, in [1]). This finding that the resistance to crack growth is dependent on crack length, until a fully developed-process-zone (FDPZ) is established, is based on the experimental evidence. With the development of an FDPZ, at least a portion of the crack is open and traction-free. Subsequent crack growth is increasingly controlled by the bulk material behaviour, not the separation mechanisms in the process zone. As just noted, the non-universal nature of the tensile fracture of sea ice was first published in 1996 [1]. This has not stopped the publication of universal scaling laws for sea ice from grain scale to geophysical scale. The non-universal scaling discussed in [1], along with the additional evidence provided in this paper, signifies that there might possibly be an uninterrupted scaling law between, say, of the order of meters to geophysical scale, but definitely not between grain scale to geophysical scale.

## 3. Creep compliance of sea ice

The reality of the tensile fracture of a single crack in ice is that the creep and fracture deformations are coupled. The form chosen for the creep compliance of the bulk material is motivated by laboratory-scale uniaxial compressive experiments [13] on saline ice, and the observation [14] that given a relatively low compressive stress (0.5 MPa) imposed at −10°C, the creep strain varies approximately as *t*^1/2^, namely,
3.1
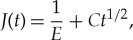


in which *E* is the short-time modulus that can be obtained from the initial slope of the load-crack mouth opening displacement plot and *C* is as yet left unspecified. The truncated fractional power-law form of the creep compliance in (3.1) corresponds to a three-parameter fractional linear solid [15].

## 4. Viscoelastic fictitious crack model

To analyse sea ice fracture tests in the presence of coupled creep and fracture deformations, it proved necessary to first develop a viscoelastic-fictitious-crack-model (VFCM) [16]. The basis for this approach is the fictitious crack model (FCM) [17] as well as the creep compliance function in (3.1).

**Figure 2.**
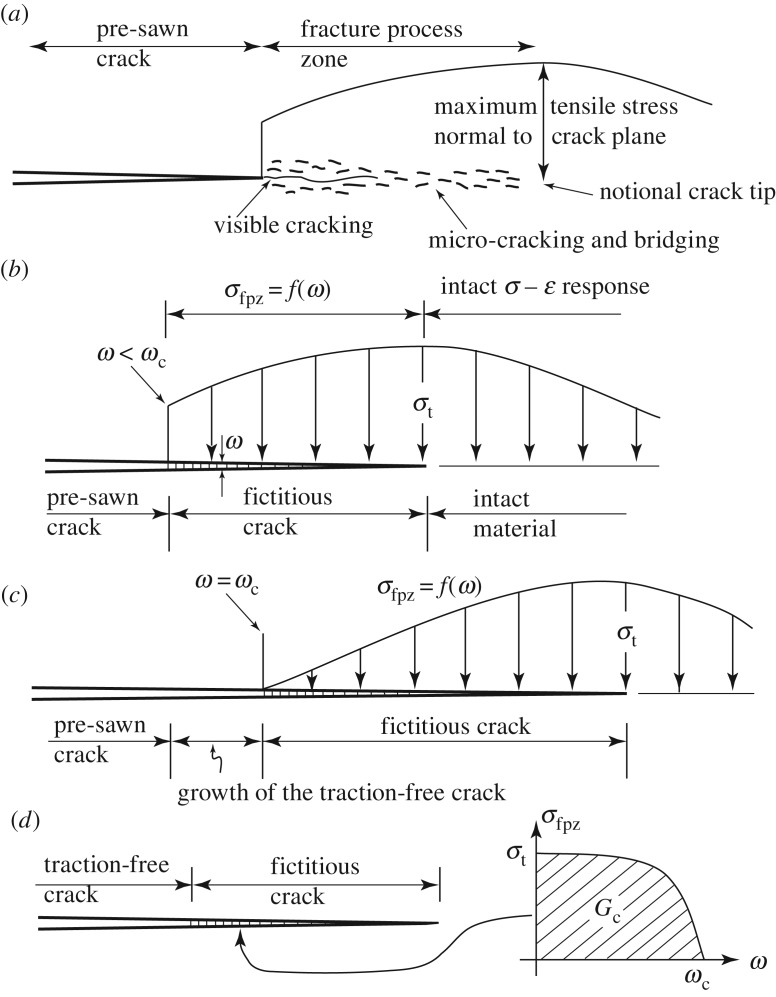
Fictitious crack model: (*a*) the physical loaded through-the-thickness crack in sea ice; (*b*) stress distribution in FPZ before, and (*c*) during, growth of the traction-free crack; (*d*) stress-separation curve (this figure is a duplicate of fig. 2 in [18]).

In its unloaded state, the fabricated (sawn) crack may be viewed as having a well-defined crack tip with a point-sized process zone. On being loaded, this through-the-thickness crack has no well-defined traction-free crack tip (figure 2*a*) but rather may be viewed as a traction-free crack attached to a fracture process zone, within which the localized micro-cracking, bridging and micro-crack coalescence accumulates at the same time as (normal to the crack plane) the tensile closing stress decreases (while the separation of two adjacent points on either side of the crack plane increases). Before the application of an opening load, the effective crack length may truly be taken equal to the pre-sawn length; this is not true once the load has been applied. Loading causes micro-cracking and bridging, and the effective crack length increases. In the VFCM, the separation normal to the crack plane due to the micro-cracking activity (not including deformation due to the straining of intact ligaments) is all assumed to occur solely on the crack plane (ahead of the traction-free crack).

In other words, the process zone is assumed to have zero width. The tensile stress carried at a particular point within the FPZ (*σ*_fpz_) is assumed to be a function of the separation *w* of the FPZ at that point (figure 2*b*). As the traction-free crack advances (figure 2*c*), points lying within the fractured zone experience first the ability to transmit the full tensile strength, but as the one point becomes two points which then separate, the stress transmitted softens and is a function of the separation *w*. At some critical separation *w*_c_, no tensile stress can be transmitted (figure 2*c*). The particular shape of this stress-separation-curve (SSC) (figure 2*d*) is regarded as a material property: the energy absorbed per unit crack area advance (*G*_c_) of the traction-free crack is represented by the area under this curve. Given that no well-defined crack tip exists, the notional crack tip is assumed to coincide with the point ahead of the traction-free crack at which the tensile stress normal to the crack plane is equal to the local tensile strength *σ*_t_. The FPZ lies between the traction-free crack tip and the notional crack tip and this FPZ is often called a *fictitious crack*. Within the fictitious crack, the cohesive stress *σ*_fpz_ and the opening of the crack faces *w* are related by the *σ*_fpz_ − *w* curve (figure 2*d*).

**Figure 3.**
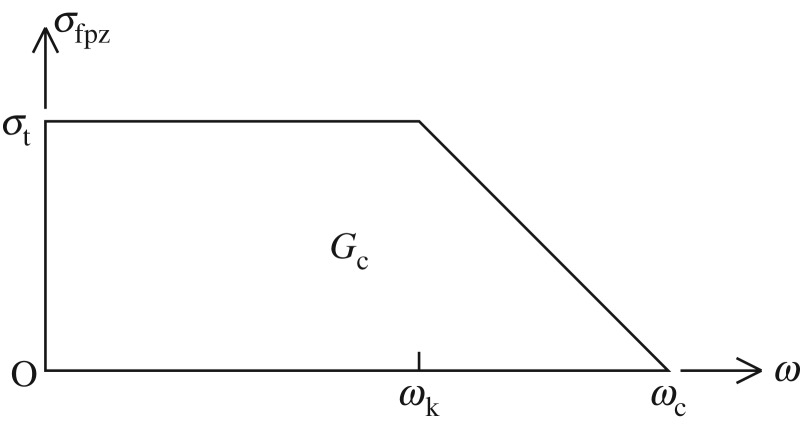
Generic stress separation curve for sea ice.

The response of material points lying outside of the FPZ is governed by the applicable *σ* − *ε* behaviour of the bulk material. Only if the FPZ is very small with regard to the specimen size is the one-parameter Griffith LEFM theory applicable. Assuming that the size (*A*_fpz_) of the fictitious crack is not negligible, knowledge of the closure stress function *σ*_fpz_(*w*) is essential. An adequate VFCM for sea ice thus requires the SSC: *σ*_t_, *w*_k_, *w*_c_ and *G*_c_ (see figure 3).

For any sea ice fracture test, the SSC as well as the viscoelastic characterization of the sea ice is not known. This information must back-calculated such that predicted results match the experimental results. The SSC is assumed to be dependent on the separation distance as well as on the rate of separation and the bulk material is considered to be linearly viscoelastic. The procedure used to carry out the match between the model and experiment is shown as a flow-chart in [15] (see fig. 5 therein). The computations were initiated by assuming a Dugdale-type of stress-separation curve followed by a linear stress-separation curve. It was found that none of these two simple approximations could predict all the experimental observations. To improve the approximations further, bilinear, trilinear, etc., shapes of the stress-separation curve were tried. Each shape tried was kept between the linear and the Dugdale shape, which had formed the bounds. The whole procedure is considered converged when the following criteria are satisfied to a reasonable accuracy: (1) the CMOD and COD as a function of time are predicted correctly; (2) the CTOD as a function of time is predicted correctly; (3) the peak load reached is same as the experimental peak load and is reached at the experimentally observed time; (4) the CTOD at macroscopic crack growth is predicted correctly and occurs at the experimentally observed time. The initial slopes of the load-CMOD and load-CTOD curves are sensitive to the initial slope of the SSC and the magnitude of *σ*_t_. Once the initial slopes of the computed and experimental load versus CODs have been matched, then the remaining shape and slope of the SSC was influenced more by the consideration that the point of instability must occur at the experimentally obtained CTOD.

No well-defined crack tip is presumed to be physically identifiable, other than the assumption that it coincides with a position at which the combined SIF is zero. The stress normal to the crack plane at this position is equated with the local tensile strength *σ*_t_, as portrayed in figure 2. Should sub-size fracture tests be conducted under load control, the FPZ will detach from the fabricated traction-free crack tip at peak load while the magnitude of the CTOD at the trailing edge (at *X* = *A*_te_) of the FPZ *w* < *w*_c_ and *σ*_ fpz_ > 0, noting that *σ*_coh_ = 0 only for *w*≥*w*_c_. The length of the process zone *A*_fpz_ is not assumed *a priori* to be small in comparison with the size of the test specimen. As portrayed in figure 2, the nonlinear processes associated with the cracking of sea ice are assumed to be crowded near the crack plane ahead of the crack tip. All of these deformations are collected on the crack line ahead of the fabricated crack.

The growth of the process zone is governed by the interaction of an externally applied crack face pressure *σ*_app_ and the cohesive stress *σ*_coh_ acting over *A*_te_ < *X* < *A*, in which *A* = *A*_te_ + *A*_fpz_. At the notional crack tip *X* = *A*, the combined SIF is specified to be zero. That is, *K*_total_ = *K*_app_ + *K*_coh_ = 0 at *X* = *A*, whilst the crack opening displacement is obtained by superposition: *w* = *w*_app_ + *w*_coh_.

In the VFCM [16], the bulk material behaviour is modelled as linearly viscoelastic. More specifically, for an ENSP with the crack loaded by the pressure *p*(*x*, *t*), with *x* = *X*/*L*, *a* = *A*/*L*, the viscoelastic COD is given by
4.1
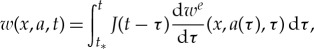
in which *J*(*t*) is the creep compliance, and *w*^*e*^ = *w*^*e*^_app_ + *w*^*e*^_coh_, with
4.2
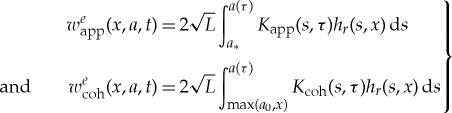
and
4.3
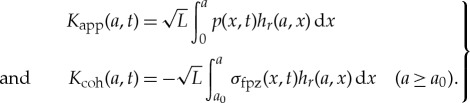
The weight function *h*_*r*_(*a*, *x*) is treated in detail in [19]. Many additional details regarding the solution procedure for the VFCM outlined above are detailed in [15,16].

Stated in words, the COD comprises a viscoelastic component due to the bulk material, as well as a fracture component due to localized softening in the cohesive zone. In the VFCM, the size-independent shape of even the rate-independent SSC is unknown at the outset, as is the exact form of the creep compliance function that must be used to analyse the *in situ* full thickness first-year sea ice fracture tests completed during the Phase II, A2 and A3 field trips. These issues are coupled. The solution procedure must back-calculate both the SSC and the creep compliance function. The back-calculation procedure is explained in detail in [15,16].

The accuracy and uniqueness of the above back-calculations were addressed in §10 of [20] for the case of an isolated cohesive crack in tension. In this analytical study, by which an approximate solution is obtained, linear softening is prescribed at three discrete locations: at the traction-free crack-tip and at two intermediate locations in the cohesive zone. The analogue, experimentally, is that the COD is measured at three locations. In §5 of [20], the linear softening integral equations were solved via a piecewise linear approximation, with a study of convergence, to establish accuracy to four significant digits. The linear softening results presented in §5 are regarded as *exact*. The discrete three COD approximate solution is very accurate. The maximum error in the fracture energy, for any traction-free crack length (*A* in figure 1), is 0.15%; the normalized process zones differed by 7 × 10^−4^ (as pointed out in more detail in [20]).

In the A3-SP1, A3-SP2 and A3-SP3 *in situ* fracture tests the COD was measured at five, seven and six locations, respectively, along the fabricated crack and ahead of the crack where the cohesive process zone would form. Based on the above investigation in §§5 and 10 of [20], the accuracy of the back-calculations reported in this paper should be formidable.

## 5. Arctic sea ice: Resolute, NWT, 1993

In the early nineties, the influence of test specimen size on the tensile fracture behaviour of sea ice was targeted as a key research topic. During Phase II, a sequence of laboratory- to structural-scale *in situ* full thickness (*h* = 1.8 m) load and crack-mouth-opening-displacement (CMOD) control fracture tests were conducted on first-year sea ice at Resolute, NWT, using self-similar (plan view) ENSPs (*A*/*L* = 0.3) [21]: 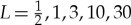 and 80 m. The sea ice salinity *S*_i_, density *ρ*_ i_, temperature *T* and grain size *d* all varied with depth in the ice sheet at Resolute, NWT [22]. The tests were conducted between April 17 and May 8 in 1993; during this time period, the ice temperature at the ice surface increased from −17°C to −12°C. The average grain size *d*_av_ was approximately 15 mm. With a size range of 1 : 160, the data obtained were used, via different size effect analyses, to evaluate the influence of scale effects on the fracture behaviour of sea ice over the range 10^−1^ m (laboratory) to 100 m and to predict the scale effect on tensile strength up to 1 km [1]. The stress-separation-curves (SSCs) were back-calculated [15] as a function of the specimen test size. Clearly, for the plan-view two-dimensional self-similarity that was maintained (with *A*/*L* = 0.3), the smaller test sizes 0.5 × 0.5 × 1.8 m^3^ and 1 × 1 × 1.8 m^3^ had very low aspect ratios (*L*/*h*), with *h* being the ice thickness. Even for the test size *L* = 3 m, the aspect ratio is low; however, the SSC determined at the point of incipient crack growth for *L* = 3, 10, 30 and 80 m was invariant. The SSC was convex, bilinear, with *σ*_t_ = 0.5 MPa, *w*_k_ = 20 μm, *w*_c_ = 40 μm and *G*_c_ = 15 J m^−2^ (figure 3).

Regarding polycrystallinity, the requisite number of grains spanned by the transitional test size proved to be 200 [23], much larger than the number 15 typically quoted for regular tension-compression testing [24]. The size-independent fracture energy proved to be 15 J m^−2^ [15], this value also significantly larger than the 1 J m^−2^ still being often quoted in the literature. The requisite LEFM test size for the edge-cracked square plate geometry (for loading durations of less than 600 s and an average grain size of 1.5 cm), was found to be greater than 3 m [23]. Size effect analyses of sub-ranges of the data showed that unless the specimen sizes tested were themselves sufficiently large, the true nature of the scale effect was not revealed. The associated fracture process zone sizes for *L* = 3, 30 and 80 m were determined to be *A*_pz_ = 120, 160 and 100 mm, respectively. The short-time modulus for the last two sizes were determined to be given by *E*_30_ = 6.4 GPa and *E*_80_ = 4.7 GPa. The smaller short-time elastic modulus for the 80 m test size is a reflection of a more compliant specimen which results in an earlier termination of the process zone development. The role played by the short-time elastic modulus is significant, as revealed by the fact that *A*^30^_fpz_∝*A*^80^_fpz_ × *E*_30_/*E*_80_. The greater compliance for the 80 m test size is caused by a slightly slower loading rate: 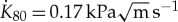 while 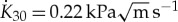.

The edge-notched-square-plates (ENSPs) tested at Resolute were tested in load or CMOD control. Two-dimensional self-similarity was maintained (with *A*/*L* = 0.3). In load control, unstable cracking ensued at peak load, with the stable development of the fracture process zone (FPZ) terminated at that point. A size effect analysis [25] confirmed that the FPZs developed in the critical size region (0.5 < *L* < 3) also scaled self-similarly. A key piece of information obtained from the tests at Resolute was that the transitional test size was of order 3 m. The other significant finding was that the VFCM could model the fracture experiments, with an invariant SSC being obtained for the tests at and above 3m. The times to peak load in the tests at Resolute were of order 500 to 1000 s; however, the loading rate was not well controlled. Load control meant that the rate of crack growth dΔ*a*/d*t* was not uniform, especially as the FPZ started to grow in size, and crack-growth-instability became imminent. At that time questions remained as to whether the fracture behaviour would vary significantly with load control versus displacement control, and controlled loading rate tests.

The above introduction summarizes the state of knowledge prior to the Antarctic sea ice fracture tests that are now described.

## 6. Antarctic sea ice: McMurdo Sound, 2001 and 2004

**Figure 4.**
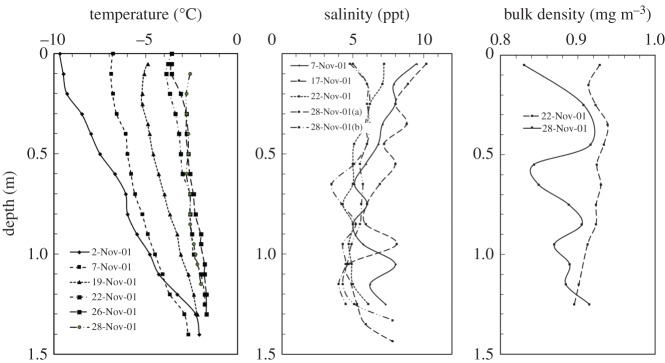
The temperature, salinity and density variation with depth for field trip A2.

**Figure 5.**
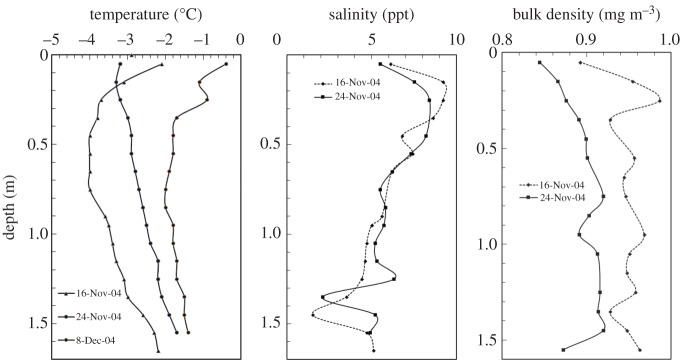
The temperature, salinity and density variation with depth for field trip A3.

These ENSP *in situ* experiments were conducted on McMurdo Sound offshore from Cape Barne, Ross Island (2001: S 77°35.033^′^, E 166°06.005^′^; 2004: S 77°29.367^′^, E 165°17.442^′^). The 2001 (2004) field trip is identified as A2 (A3). Each first-year sea ice sheet encountered was predominantly congelation ice with a top layer of granular ice, with evidence of platelet ice at depth. The particular test configurations to be discussed include A2-SP2, A2-SP8, A3-SP1, A3-SP2 and A3-SP3. The A2-SP2 tests were conducted on 14–15 November 2001 with a test size of 5 × 5 × 1.42 m^3^; A2-SP2's test configuration is portrayed in fig. 1 of both [18,26]. The A2-SP8 tests were conducted on 26 November 2001 with a test size of 10 × 10 × 1.42 m^3^; A2-SP8's test configuration is portrayed in fig. 1 of [27]. The A3-SP1, A3-SP2 and A3-SP3 tests were conducted on 1, 3 and 4 December 2004, respectively. In A3, the ENSP test size was 5 × 5 × 1.66 m^3^, and the A3-SP1 test configuration is portrayed in figure 1. The temperature, salinity and density profiles for the A2 and A3 field trips are shown in figures 4 and 5.

Floating first-year sea ice naturally exists with significant temperature and salinity gradients, and less so for bulk density. Additionally, the individual grains that make up an ice sheet can become crystallographically aligned due to under-ice currents, which generates anisotropy in both the fracture and constitutive response. The fracture and constitutive behaviour of sea ice are functions of these complex physical properties (to include the above-mentioned flaw structure) and thermal state.

The creep compliance constant *C* introduced in §3 is empirical and does not naturally account for physical and microstructural characteristics of the ice sheet being fractured. Its time dependence, however, is based on laboratory experiments and it adequately represents the creep behaviour of sea ice for small strains. It is a useful representation for back calculations since the aggregate effect of thermal and material properties gradients is captured in a single creep-compliance constant. For the eventual forward calculations, by contrast, a mechanistic formulation that explicitly employs the physical properties [28] will be employed. To understand and eventually model the mechanical behaviour in terms of these properties, profiles of the type seen in figures 4 and 5 were acquired for each set of field experiments.

The physical properties and microstructure of the 2001 ice sheet, the cyclic-loading response, and the acoustic emission activity from an extensive series of experiments conducted on A2-SP2 of the *in situ* test specimens are described in [29,30]. The testing of A2-SP2 comprised 15 separate tests, many of these tests targeting the cyclic behaviour of this first-year sea ice. Varying the cyclic-loading frequency and amplitude provided a means to examine rate effects on the anelastic and viscous components of strain and the extent of micro-cracking near the crack tip. The viscous deformation rate estimated from the experiments exhibited an increasing power-law exponent with values between one and three. Acoustic emission monitoring indicated that micro-cracking occurred in a process zone near the crack tip, and the size of the process zone increased with decreasing cyclic loading frequency. In particular [30], micro-cracking occurred in advance of the propagation of the starter crack, and the brine inclusion structure was seen to influence the macroscopic crack path; micro-cracking occurred in a region surrounding the stationary crack tip, while the number of acoustic events exhibited a steep fall-off with distance from the crack tip, and the micro-cracking activity moved with the crack tip during crack propagation.

## 7. The cracking behaviour

**Figure 6.**
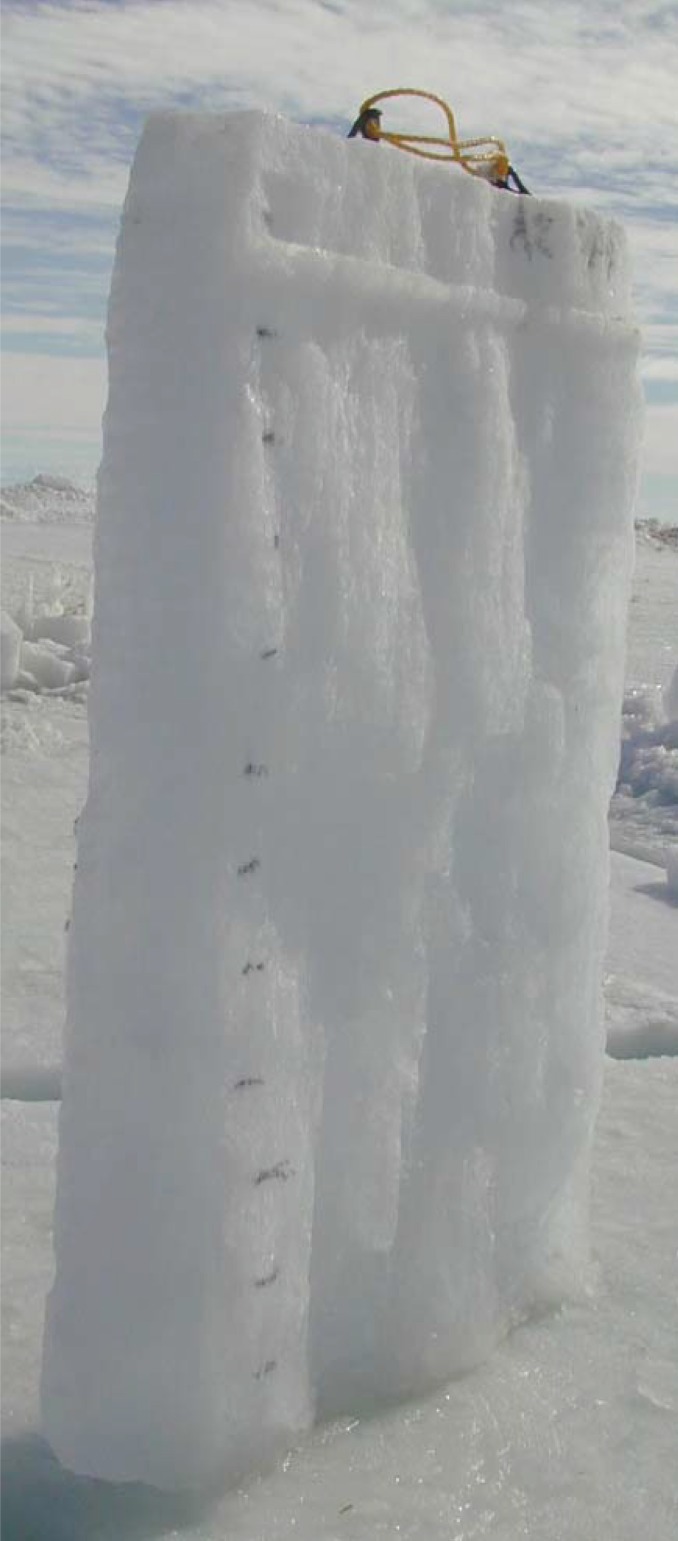
Top-to-bottom slice of test specimen A2-SP9: note the tortuosity of the crack face. (Online version in colour.)

**Figure 7.**
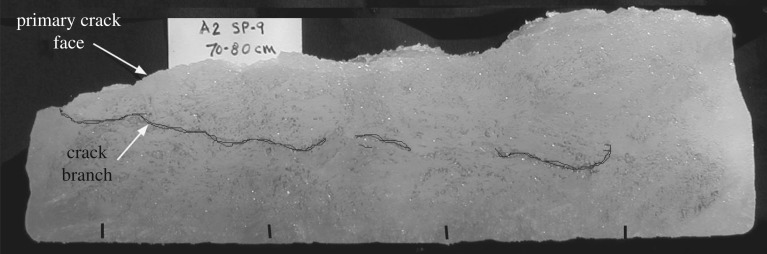
Test specimen A2-SP9: crack growth is a multiple crack path competitive process.

Sea ice contains many inhomogeneities such as brine pockets, brine drainage channels, as well as stress concentrators such as grain boundary junctions. Near the crack tip, deformation modes include grain boundary sliding, grain boundary separation and micro-cracking. The crack does not propagate as a vertical straight line. Rather, it seems to propagate in sequential bottom-top process, while seeking out nearby out-of-plane zones weakened by the presence of brine drainage channels, which leads to rather tortuous crack paths, as shown in figure 6. The tortuosity is itself reflective of crack growth accompanied by a multiple crack path competitive process (figure 7). The interpretation is that multiple, competing FPZs are attempting to get established ahead of the traction-free crack path, in addition to the FPZ that becomes the final crack path, until suddenly crack growth along the final path is found to have the lowest ERR.

## 8. The back-calculated fracture characteristics

Given the specimen size *L*, ice thickness *h*, ice sheet temperature *T* and ice sheet salinity *S*_*i*_, the VFCM data are presented in tables 2 and 3. This data include the short-term modulus *E*, the creep-compliance constant *C*, the local tensile strength *σ*_t_, the SSC-Knee-COD *w*_k_, the critical COD *w*_c_ and the critical-fracture-energy *G*_c_. Note again the generic stress-separation curve portrayed in figure 3, which is defined by the three quantities *σ*_t_, *w*_c_ and *G*_c_ (given that 2*G*_c_/*σ*_t_ = *w*_k_ + *w*_c_).

**Table 2.** Arctic Phase II-1993 and Antarctic A2-2001 field test results.

test specimen		Phase II	A2-SP2	A2-SP8
related publications		[1,3,15,16,21]	[18,26,29,30]	[27]
test date(s)		4/22/93-5/4/93	11/14-15/01	11/26/01
specimen size (square)	*L* (m)	3,10,30,80	5	10
ice thickness	*h* (m)	1.8	1.42	1.42
ice sheet temperature	*T* (°C)	−9 < *T*_av_ < − 7	−3.7 ± 1.1	−2.5 ± 0.6
salinity	*S*_*i*_ (°/_°°_)	5.0 ± 1.1	7.0 ± 1.6	5.0 ± 1.9
short-term modulus	*E* (GPa)	5.7,6.5,6.0,4.2	3.1	3.1
creep-compliance constant	*C* × 10^11^ (m^2^ N 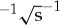 )	0.6,0.7,0.7,0.7	3.5	4.5
tensile strength	*σ*_t_ (MPa)	0.5	0.26	0.13
SSC-Knee-COD	*w*_k_ (μm)	20	20	85
critical-COD	*w*_c_ (μm)	40	80	110
fracture energy	*G*_c_ (N m^−1^)	15.0	13.0	12.7

**Table 3.** Antarctic A3-2004 field test results.

test specimen		A3-SP1	A3-SP2	A3-SP3
test date		12/1/04	12/3/04	12/4/04
specimen size (square)	*L* (m)	5	5	5
ice thickness	*h* (m)	1.66	1.66	1.66
ice sheet temperature	*T* (°C)	−2.1 ± 0.5	−2.0 ± 0.5	−1.9 ± 0.5
salinity	*S*_*i*_ (°/_°°_)	5.7 ± 1.8	5.7 ± 1.8	5.7 ± 1.8
short-term modulus	*E* (GPa)	2.8	3.1	3.4
creep-compliance constant	*C* × 10^11^ (m^2^ N 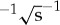 )	4.5	4.5	4.5
tensile strength	*σ*_t_ (MPa)	0.14	0.12	0.16
SSC-Knee-COD	*w*_k_ (μm)	56	70	80
critical-COD	*w*_c_ (μm)	110	100	90
fracture energy	*G*_c_ (N m^−1^)	11.6	10.2	13.6

The analyses of the Phase II, A2-SP2 and the A2-SP8 fracture tests are provided in [1,3,15,16,18,21,26,27,29,30], respectively. Space does not permit a discussion of the diverse various loading scenarios and loading rates encompassed by the data summarized in table 2. Significantly, the local tensile strength seems most affected by the ice temperature.

The back-calculated fracture characteristics for the A3-SP1, A3-SP2 and A3-SP3 tests are summarized in table 3. The associated test configurations are drawn, and the load and COD measurements are plotted in the electronic supplementary material for this paper. That information should be consulted while reading the following three paragraphs.

Test A3-SP1 comprised three separate tests. Test A3-SP1-1 involved loading under displacement control by the antecedent LVDT gage NCTOD^−^_i_. A monotonic ramp to a CMOD of 550 μm in 18.5 s was followed by unloading. A FPZ of 200 mm was introduced by Test A3-SP1-1. Test A3-SP1-2 malfunctioned. In Test A3-SP1-3, a fast ramp under load control produced growth of the traction-free in 0.2 s.

Test A3-SP2 comprised two separate tests, each involving displacement control by the antecedent LVDT gage NCTOD^−^_i_. In Test A3-SP2-1, a monotonic ramp to a CMOD of 500 μm in 9 s was followed by unloading. A FPZ of 400 mm was introduced by Test A3-SP2-1. In Test A3-SP2-2, a monotonic ramp to a CMOD of 1500 μm in 4.5 s produced growth of the traction-free crack in 3 s.

Test A3-SP3 comprised three separate tests, each involving displacement control by the antecedent LVDT gage NCTOD^−^_i_. In Test A3-SP3-1, a monotonic ramp to a CMOD of 250 μm in 5 s was followed by unloading. Test A3-SP3-2 malfunctioned. In Test A3-SP3-3, a monotonic ramp to a CMOD of 2000 μm in 70 s produced growth of the traction-free crack in 5 s.

In [31], it was found that the tensile strength of isothermal first-year sea ice cylinders decreased rapidly at ice temperatures warmer than −5°C; for the two strain rates studied in [31] (10^−5^ s^−1^ and 10^−3^ s^−1^), the strain rate was not a factor. In table 3, the influence of ice temperature is even stronger than that found in [31]: the *in situ* tensile strength is decreasing more rapidly with ice temperature. Remember that the tests here are *in situ*, not tensile tests on cores that have been frozen, stored, then warmed back up (which is when further brine drainage occurs).

The final test for A3-SP1 was conducted under load control; the final tests for A3-SP2 and A3-SP3 were conducted under displacement control, and were 25 and 15 times slower, respectively, than that for A3-SP1. As in [31], however, loading rate was not a significant factor. Nor was the method of load control.

## 9. Conclusion

During the Phase II field program, 15 separate *in situ* experiments were completed on 15 floating square plates of ice with sizes 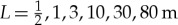, the number of tests at each size being 2, 2, 3, 2, 5, 1, respectively. Ten of these tests were conducted under load control and five under displacement control. Fifteen separate *in situ* cyclic and fracture tests were conducted on test plate A2-SP2. Many of these tests targeted solely cyclic behaviour as well as the cracking induced by cyclic behaviour. Four separate tests were conducted on test plate A2-SP8, with the last test concentrating on stable fracture of the whole sample. One esoteric feature of the study in [27] is the finding that the FPZ grew stably during the unloading portion of a cycle. The test plates A3-SP1, A3-SP2 and A3-SP3 were subjected to two separate tests each. All of these different loading and cracking scenarios have been analysed by the VFCM.

After considerable detailed analysis, it is clear that the ice temperature is very important, while the loading rate, apparently, is not a significant factor. As the ice temperature becomes warmer (especially above −5°C), there is a rapid decrease in the local tensile strength, while the critical separation *w*_c_ increases substantially. Significantly, the overall critical-ERR is not varying much at all, indicating slightly decreased values for warmer tests.

At a given ice temperature and salinity, the tensile fracture characteristics of a single crack in first-year sea ice will vary with scale (test size) *L*, initial crack length *A* (another scale), test methodology (load control or displacement control) [20], until the test sizes are of the order of several metres, and the cracks are of the order of a metre in size. With the all-important availability of the SSC, the fracture behaviour can be predicted by the VFCM (as demonstrated in [23]).

The VFCM introduced in [16] can handle crack formation from an un-notched state, crack propagation from small to large, crack growth under load control (for which the process zone will steadily increase with increasing crack length, but never become fully developed), or crack growth under displacement control (for which the process zone will steadily decrease, and a FDPZ is observed). This VFCM is required since cracks in sea ice nucleate and grow on different planes, on many orientations, from un-cracked surfaces, from within, and propagate from existing crack tips.

## Supplementary Material

Supplementary Information

## Supplementary Material

Fig. S4a

## Supplementary Material

Fig. S4b

## Supplementary Material

Fig. S5a

## Supplementary Material

Fig. S5b

## Supplementary Material

FigS6a

## Supplementary Material

Fig. S6b

## Supplementary Material

Fig. S7a

## Supplementary Material

Fig. S7b

## Supplementary Material

Fig. S8a

## Supplementary Material

Fig. S8b

## Supplementary Material

Fig. S9a

## Supplementary Material

Fig. S9b

## Supplementary Material

Supp. Info. MATLAB figure Names and Captions

## Data accessibility

The data for the Arctic Phase II field trip have been published in [1,3,15,16,21]. The data for the A2-SP2 field test have been published in [18,26,29,30]. The data for the A2-SP8 field test have been published in [27]. The data for the A3-SP1, A3-SP2 and A3-SP3 field tests are provided in the electronic supplementary material.
