# Supplementary material for: Tensile fracture of a single crack in first-year sea ice
Source: Philos Trans A Math Phys Eng Sci. 2018 Aug 20;376(2129):20170346. doi: 10.1098/rsta.2017.0346 (PMC6107610; doi:10.1098/rsta.2017.0346)
Supplement: Supplementary Information [file rsta20170346supp1.pdf]

SUPPLEMENTARY INFORMATION

J.P. Dempsey **DOI: 10.1098/rsta.2017.0346**

The A3-SP1, A3-SP2, and A3-SP3 Test Configurations

The A3-SP1, A3-SP2 and A3-SP3 test configurations are shown in Figures S1, S2 and S3, respectively. All dimensions are in cm. The LVDT ranges ($\pm$ range) are in microns. Both an 'on the ice surface' ($_{\mathrm{i}}$) and an 'up in air' ($_{\mathrm{u}}$) gage were used to record the crack-mouth-opening-displacement (CMOD), the intermediate-crack-opening-displacement (COD), and the antecedent near-crack-tip-opening-displacement ($NCTOD^-$). LVDT gages were also placed 10 cm ahead of crack tips to record the crack-tip-opening-displacement ahead the crack tip ($NCTOD^+$). So-called 'fictitious' gages (F1, F2, F3) were placed yet further ahead of the crack tip to identify the crack-opening-displacement as the process zone advances. All measuring stations located ahead of the crack tip ($NCTOD^+$, F1, F2, F3) included both a 'coarse range' ($_{\mathrm{c}}$) and a 'fine range' ($_{\mathrm{f}}$) gage. In Figures S1, S2 and S3, the small dark circles identify the LVDT locations. Side views of the pre-sawn crack are shown in Figures S1b, S2b and S3b, respectively. Cracks were initially cut to about 250 cm by a specially constructed ice saw, and then sharpened with a long hand-held ice saw. Load was applied to the specimen using a flatjack loading device (a flatjack here being a thin stainless-steel bladder). Each flatjack was pressurized in these tests with nitrogen gas. A servovalve was used to control the flatjack pressure, which allowed tests to be performed in both load and displacement control. The flatjacks were positioned as shown in Figures S1b, S2b and S3b, respectively.

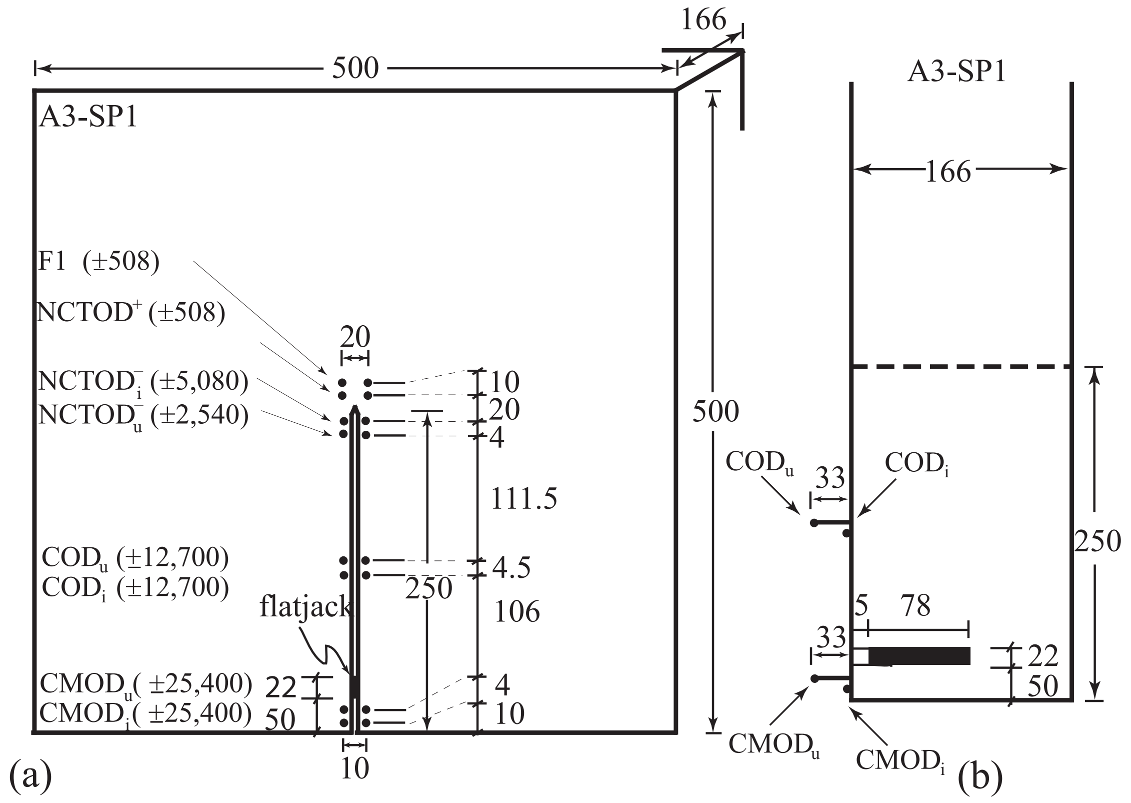

Figure S1: a) Plan view of the test configuration for A3-SP1; b) Side view of the pre-sawn crack showing the flatjack placement. All dimensions are in cm; the LVDT ranges (± range) are in microns.

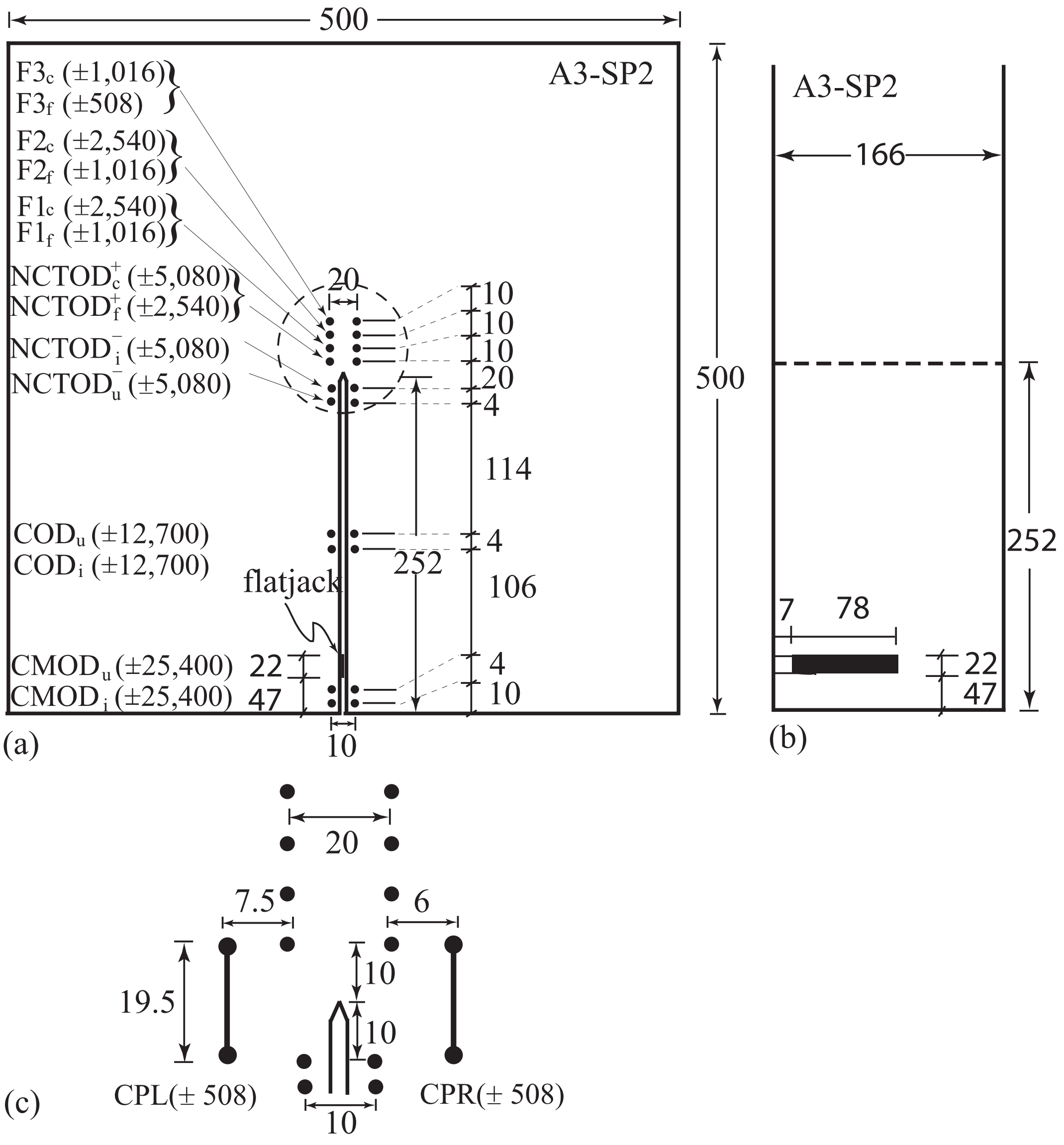

Figure S2: a) Plan view of the test configuration for A3-SP2; b) Side view of the pre-sawn crack showing the flatjack placement. c) Closer look at LVDT locations ahead of the crack tip. All dimensions are in cm; the LVDT ranges (± range) are in microns.

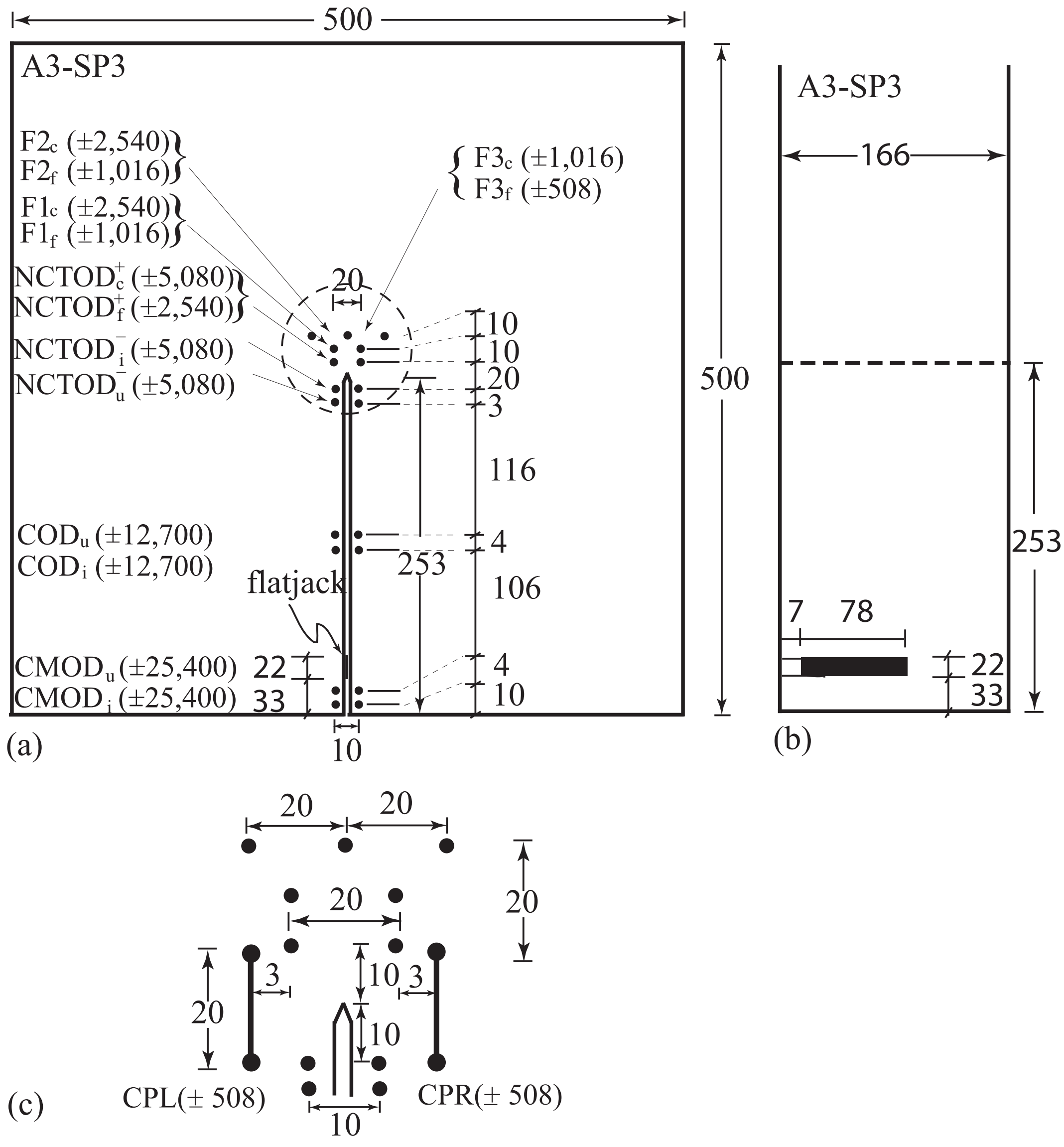

Figure S3: a) Plan view of the test configuration for A3-SP3; b) Side view of the pre-sawn crack showing the flatjack placement. c) Closer look at LVDT locations ahead of the crack tip. All dimensions are in cm; the LVDT ranges (± range) are in microns.

A3-SP1 Load and COD Measurements

Test A3-SP1 was conducted on December 1, 2004 and comprised of 3 separate tests. Test #1 was conducted under displacement control of the antecedent LVDT gage $\mathrm{NCTOD}_{\mathrm{i}}^{-}$. The crack opening displacement and the flatjack pressure response versus time are plotted in Figure S4. Considerable noise was recorded by all LVDT gages. One LVDT gage ($\mathrm{NCTOD}_{\mathrm{u}}^{-}$) even showed noise of magnitude 200μm. As shown in Figure S4b, a nonzero load started at $t = 7.3$s. The peak load occurs at $t = 25.81$s with flatjack pressure 0.316 MPa following by a reasonable rate of unloading. Crack opening displacements all reach peak value around $t = 26$s, but could not be located that accurately due to the noise. In Test #2, the test was shut down after 24 seconds without recording any useful data. In Test #3, no attempt was made to utilize computer controlled loading. Rather, the handle on the nitrogen bottle was quickly lowered to attain the fastest possible loading without any COD control, the flatjack ballooned and the specimen abruptly and completely fractured. The associated results are portrayed in Figure S5. The gage $\mathrm{NCTOD}_{\mathrm{u}}^{-}$ gage did not work well in this test. As shown in Figure S5b, a the loading started at $t = 6.9$s.

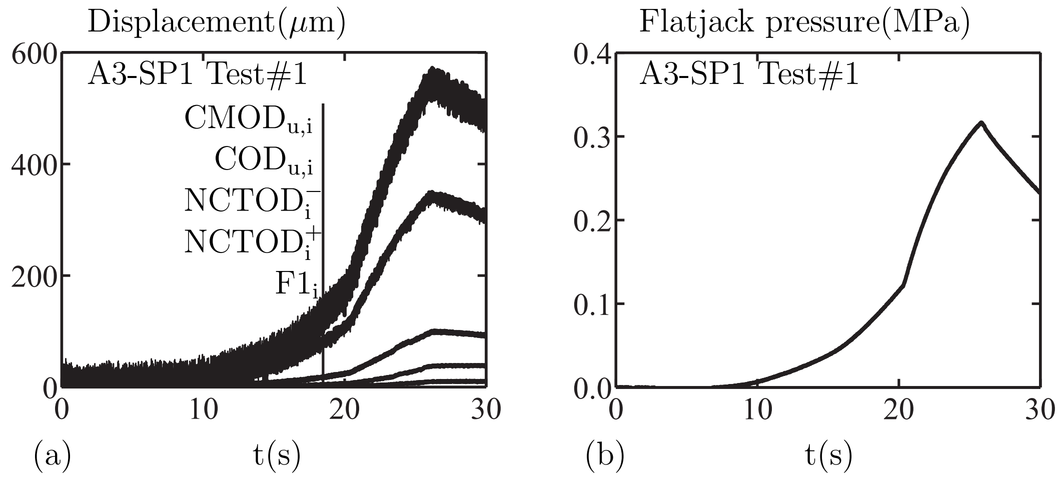

Figure S4: A3-SP1 Test #1(a) Crack opening displacement; (b) Flatjack pressure.

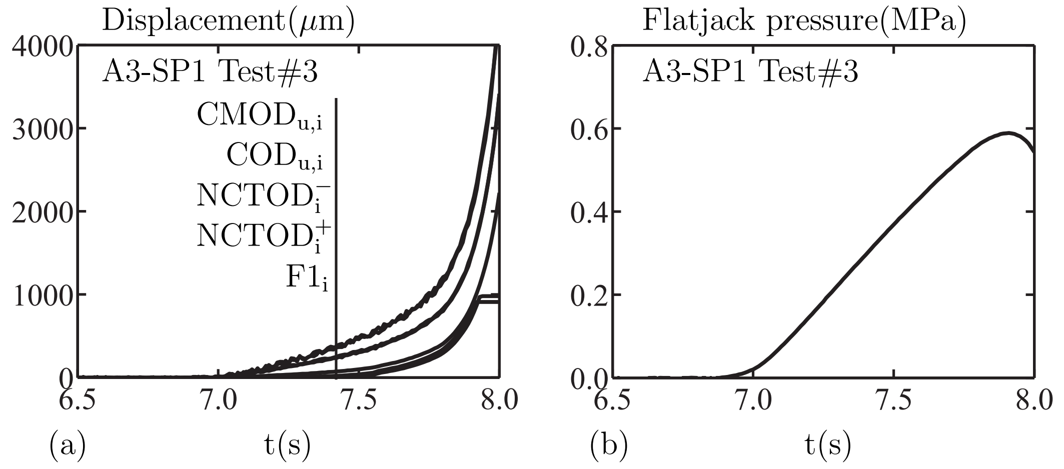

Figure S5: A3-SP1 Test #3(a) Crack opening displacement; (b) Flatjack pressure.

A3-SP2 Load and COD Measurements

Test A3-SP2 was conducted on December 3, 2004 and was comprised of 2 separate tests. Both Test #1 and #2 was conducted under displacement control of the antecedent LVDT gage $\mathrm{NCTOD}_\mathrm{i}^-$. Gage readings of Test #1 were disturbed by a lot of scatter. Plots of deformation and load gage readings versus time of Test #1 are shown in Figure S6. As shown in Figure S6b, a the loading started at t = 21s. The peak load occurs at t = 30s with flatjack pressure 0.33 MPa. In Test #2, the objective was to stably fracture the 5 m square test piece in a relatively fast loading rate. The associated results are plotted in Figures S7. As shown in Figure S7b, the loading started at t = 4.9s. The traction-free crack growth initiation occurred between t = 8.3s and t = 8.6s for Test #2 of A3-SP2.

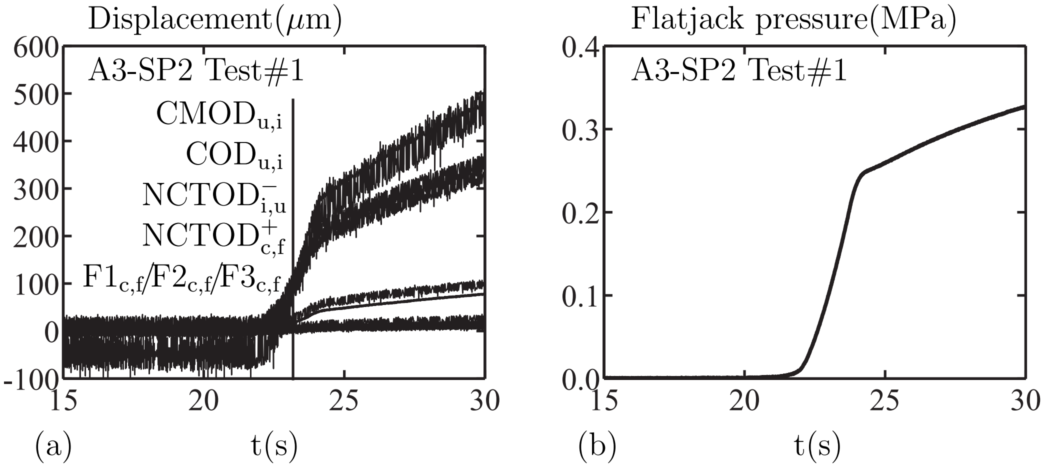

Figure S6: A3-SP2 Test #1(a) Crack opening displacement; (b) Flatjack pressure.

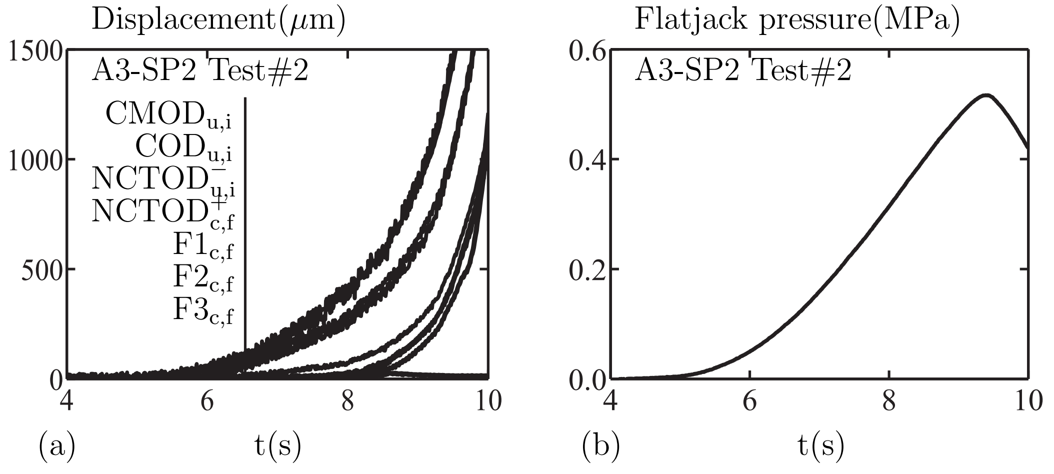

Figure S7: A3-SP2 Test #2(a) Crack opening displacement; (b) Flatjack pressure.

A3-SP3 Load and COD Measurements

Test A3-SP3 was conducted on December 4, 2004 and comprised of 3 separate tests. All three tests were conducted under displacement control of the antecedent LVDT gage $\mathrm{NCTOD}_{\mathrm{i}}^{-}$. The crack opening displacement and the flatjack pressure response of Test #1 versus time are plotted in FigureS8. Gage readings of Test #1 were disturbed by considerable noise. In Test #2, the test was shut down after 18 seconds without recording any useful data. In Test #3, the objective was to stably fracture the 5 m square test piece at a relatively slow loading rate. The associated results are plotted in Figure S9. As shown in Figure S9b, a the loading started at $t = 31$s. and reached the first peak load 0.5 MPa at $t = 39$s. The traction-free crack growth initiation occurred between $t = 37.5$s and $t = 38$s for Test #3 of A3-SP3.

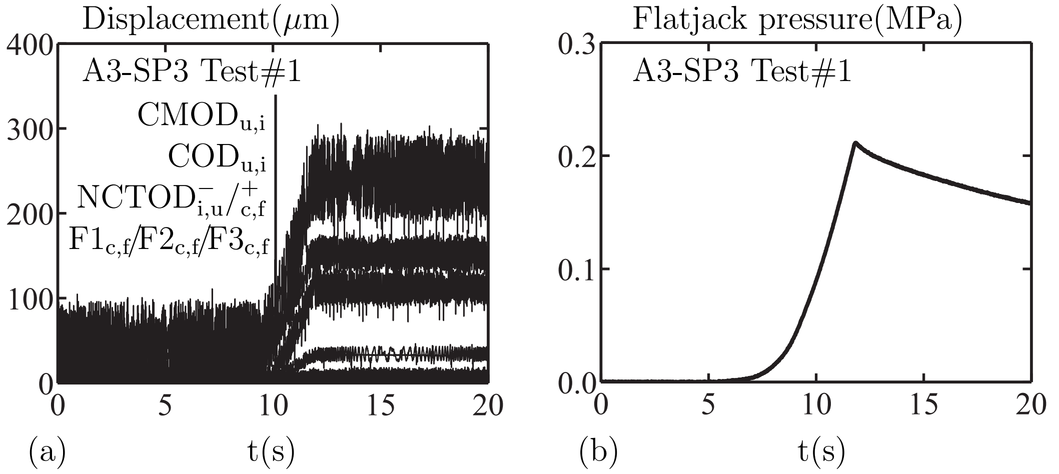

Figure S8: A3-SP3 Test #1(a) Crack opening displacement; (b) Flatjack pressure.

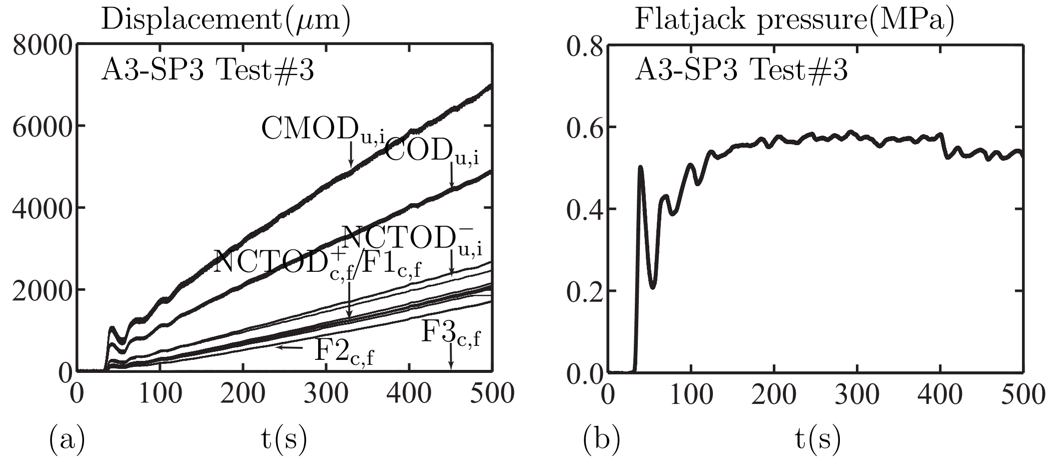

Figure S9: A3-SP3 Test #3(a) Crack opening displacement; (b) Flatjack pressure.
