# Supplementary material for: Tensile fracture of a single crack in first-year sea ice
Source: Philos Trans A Math Phys Eng Sci. 2018 Aug 20;376(2129):20170346. doi: 10.1098/rsta.2017.0346 (PMC6107610; doi:10.1098/rsta.2017.0346)
Supplement: Supp. Info. MATLAB figure Names and Captions [file rsta20170346supp14.pdf]

Supplementary Information File Names and Captions

dempseyA3SupplementaryInformation.pdf

This document portrays the A3-SP1, A3-SP2 and A3-SP3 test configurations, the crack opening displacement versus time, and flatjack pressure versus time for the six tests: A3-SP1 Tests #1,#3; A3-SP2 Tests #1,#2; A3-SP3 Tests #1,#3.

MATLAB figure	caption
**dempseyA3FigS4a.fig**	A3-SP1 Test#1 crack opening displacements versus time
**dempseyA3FigS5a.fig**	A3-SP1 Test#3 crack opening displacements versus time
**dempseyA3FigS6a.fig**	A3-SP2 Test#1 crack opening displacements versus time
**dempseyA3FigS7a.fig**	A3-SP2 Test#2 crack opening displacements versus time
**dempseyA3FigS8a.fig**	A3-SP3 Test#1 crack opening displacements versus time
**dempseyA3FigS9a.fig**	A3-SP3 Test#3 crack opening displacements versus time

MATLAB figure	caption
**dempseyA3FigS4b.fig**	A3-SP1 Test#1 flatjack pressure versus time
**dempseyA3FigS5b.fig**	A3-SP1 Test#3 flatjack pressure versus time
**dempseyA3FigS6b.fig**	A3-SP2 Test#1 flatjack pressure versus time
**dempseyA3FigS7b.fig**	A3-SP2 Test#2 flatjack pressure versus time
**dempseyA3FigS8b.fig**	A3-SP3 Test#1 flatjack pressure versus time
**dempseyA3FigS9b.fig**	A3-SP3 Test#3 flatjack pressure versus time
